# Frailty Is Associated with Malnutrition–Inflammation Syndrome in Older CKD Patients

**DOI:** 10.3390/nu16162626

**Published:** 2024-08-09

**Authors:** Paolo Molinari, Lara Caldiroli, Matteo Abinti, Luca Nardelli, Silvia Armelloni, Matteo Cesari, Giuseppe Castellano, Simone Vettoretti

**Affiliations:** 1 Unit of Nephrology, Dialysis and Kidney Transplantation, Fondazione IRCCS Ca’ Granda Ospedale Maggiore Policlinico di Milano, 20122 Milan, Italy; paolo.molinari1@unimi.it (P.M.); matteo.abinti@unimi.it (M.A.); luca.nardelli@policlinico.mi.it (L.N.); silvia.armelloni@policlinico.mi.it (S.A.); giuseppe.castellano@unimi.it (G.C.); simone.vettoretti@policlinico.mi.it (S.V.); 2 Department of Clinical Sciences and Community Health, Università degli Studi di Milano, 20122 Milan, Italy; matteo.cesari@unimi.it

**Keywords:** frailty, malnutrition, systemic inflammation, chronic kidney disease (CKD)

## Abstract

Patients affected by chronic kidney disease (CKD) are generally considered to be frailer than those with preserved renal function. We cross-sectionally evaluated the associations between frailty, malnutrition–inflammation syndrome and circulating inflammatory cytokines in 115 older individuals with advanced CKD. As for frailty definition, we adopted Fried’s frailty phenotype (FP), while malnutrition–inflammation syndrome was assessed using the Malnutrition–Inflammation Score (MIS) and circulating inflammatory cytokines (IL-6; TNFα; MCP-1). A total of 48 patients were frail, and mean eGFR was comparable in both frail and non-frail patients (24 ± 10 vs. 25 ± 11 mL/min/1.73 m^2^; *p* = 0.63). Frail patients had higher MIS (6 [4–11] vs. 4 [3–5]; *p* < 0.0001) but cytokine concentrations were comparable in both groups. At multivariate regression, FP was independently associated with MIS, age, gender and pre-albumin but not with cytokines. However, we found some associations between inflammatory cytokines and some specific frailty criteria: weight loss and slowness were associated with MCP-1 (respectively *p* = 0.049 and *p* < 0.0001) and weakness with IL-6 (*p* = 0.005); in conclusion, in older patients with advanced CKD, frailty is strictly associated with malnutrition–inflammation syndrome but not with circulating inflammatory cytokines.

## 1. Introduction

Frailty is defined as a decrease in physiological reserves which increases vulnerability to adverse outcomes when minor stressors occur [1,2,3,4]. In older patients belonging to the general population, chronic systemic inflammation is one of the main determinants of frailty [5,6]. Chronic kidney disease (CKD) is known to be one of the most representative conditions that can accelerate premature aging [7,8]. Chronic inflammation, insulin resistance and increased uremic toxins are conditions associated with CKD that can contribute to increasing the onset of frailty in these patients [7,9,10,11]. In older CKD patients, frailty is a common condition [12,13] and its prevalence increases as renal function declines [13,14]. Furthermore, the presence of frailty increases the risk of morbidity and mortality up to twofold in patients with CKD prior to initiation of dialysis [15,16]. Frailty has also been shown to be associated with adverse outcomes, such as hospitalization and mortality, among others, in our cohort. Therefore, patients with CKD have a significantly increased risk of vulnerability, morbidity, and mortality compared to the general population due to their frequent concomitant development of frailty [17].

The onset of frailty in CKD patients has multi-factorial determinants; one of its potential causes is malnutrition [18,19]. The causes of malnutrition are complex: anorexia, changes in taste, uremic gastritis, accumulation of uremic toxins and polypharmacy may all contribute to reduce protein and energy intake [7,19,20,21]. CKD patients are also characterized by increased systemic inflammation [22] due to the accumulation of uremic toxins and pro-inflammatory mediators. This pro-inflammatory milieu may promote the onset of malnutrition–inflammation syndrome, characterized by low-grade inflammation and protein–energy wasting, which may contribute to the onset of frailty [23,24].

An unmet need in CKD patients is to identify simple and reliable clinical and laboratory markers to quantify the risk of negative outcomes, such as frailty, in order to take preventive action.

In this study, we first evaluated the associations of frailty with subclinical systemic inflammation (evaluated by circulating pro-inflammatory cytokines) and malnutrition in older individuals with advanced CKD. The aim of our study was to identify a reliable, easy-to-perform and reproducible marker for increased frailty development risk, which in turn leads to patient vulnerability and morbidity.

## 2. Materials and Methods

### 2.1. Patients and Study Design

We cross-sectionally evaluated 115 prevalent patients that attended our out-patient clinic between 9/2016 and 3/2018. All patients were selected according to the following criteria: age ≥ 65 years, CKD stages 3a to 5 in conservative therapy and relatively stable eGFR over the previous 6 months (eGFR variation lower than 2 mL/min/1.73/m^2^). eGFR was estimated according to the CKD-EPI formula [25]. We excluded patients with tumors and cirrhosis; those taking immunosuppressive drugs; those with severe heart failure (NYHA class III–IV) [26], nephrotic syndrome, thyroid diseases, bowel inflammatory diseases or infections; those who were hospitalized in the last three months; or those who were unable to cooperate. All patients were evaluated at the time of inclusion in the study and before sampling. Biochemical and urinary parameters were collected on the morning of the study visit after an overnight fast of at least 12 h. This study was approved by the Ethics Committee of our Institution (Milano 2-approval N. 347/2010) and was conducted according to the ICP Good Clinical Practices Guidelines. All patients signed their informed consent.

### 2.2. Frailty Assessment

Frailty was assessed with Fried’s frailty phenotype (FP) [27], defined by the impairment of three or more of the following items: (1) involuntary weight loss ≥4.5 kg in 12 months; (2) physical exhaustion for more than three days per week for at least three months; (3) handgrip strength <16 kg in females and <27 kg in males; (4) gait speed >0.8 m/s; and (5) reduced physical activity with a score <7 on a physical activity scale that was described elsewhere [11]. Patients who were unable to perform any of these tests due to pre-existing physical disability were excluded from this study. 

### 2.3. Body Composition and Nutritional Status

Body mass index (BMI) was determined according to the Quetelet index (kg/m^2^) [28].

Nutritional status was assessed by determining the Malnutrition–Inflammation Score (MIS), an adaptation of the SGA questionnaire that includes some objective clinical and laboratory markers relevant to CKD patients [29,30]. The MIS has been validated against other nutritional/inflammatory biomarkers and is associated with poorer prognosis in patients experiencing HD [29,30,31], peritoneal dialysis [32,33] or kidney transplantation [34] and non-dialyzed CKD patients [29]. The MIS is a score made up of 10 items, each with four levels of severity: from 0 (normal) to 3 (severely abnormal). A total score of 4–7 indicates a risk of malnutrition and a score ≥8 indicates malnourishment [29]. As the MIS is a semi-objective score and depends on the observer, the MIS was always performed by the nutritionist of our institution in order to reduce the interobserver variability.

### 2.4. Detection of Serum Levels of Cytokines

Serum cytokine concentrations were collected on the day of the visit and stored at −80 °C. Cytokine concentrations were determined using enzyme-linked immunosorbent assay (ELISA) kits according to the manufacturer’s instructions.

The following kits were used: a Quantikine ELISA Human CCL2/MCP-1 Immunoassay DCP00 human TNF-alpha ELISA Kit (Thermo Fisher Scientific, Monza, Italy). The declared sensitivity of the kit was <1.7 pg/mL for CCL2/MCP-1 and <2 pg/mL for TNFalpha. For human interleukin IL-6 (IL-6), three different ELISA kits were used and compared: human IL-6 ELISA Kit EH2IL6 (Thermo Fisher Scientific, Monza, Italy), human IL-6 Platinum ELISA BMS213/2 (Affymetrix, Thermo Fisher Scientific, Monza, Italy) and Quantikine HS ELISA human IL-6 Immunoassay HS600B (R&D Systems, Space, Milano, Italy), with sensitivities of <1 pg/mL, 0.92 pg/mL and 0.110 pg/mL, respectively. For IL-6 quantification, the results of the Quantikine HS ELISA, the Human IL-6 Immunoassay HS600B and the Human IL-6 ELISA Kit EH2IL6 were compared by a simple regression test and both results were used indifferently after establishing a significant correlation.

In each assay, the curve included zero as the last standard point. Quantikine Immunoassay Control Group 1–4 or 10 (R&D Systems, Space, Milan, Italy) equipment was used to check the acceptability of the assays. The absorbance values were measured at 450 nm using a spectrophotometer (Xenius Safas, Fontvieille, Monaco). All cytokines were assayed in duplicate.

### 2.5. Statistical Analysis

We evaluated the frequency distributions of the principal variables to test for normality. Data distributed normally were considered parametric, while data which did not show a normal distribution were considered non-parametric.

Continuous variables were reported as mean ± standard deviation (SD), for parametric data, or median with interquartile range (IQR), for non-parametric ones. Nominal/categorical variables were reported as percentages. We used Student’s *t*-test and ANOVA to compare parametric variables, while we used the Mann–Whitney “U” test or Kruskal–Wallis test for non-parametric ones.

Spearman analysis was performed to test the correlation between frailty criteria and factors associated with frailty; moreover, GLM (multivariable linear regression) was performed to test the correlations between MIS and inflammatory markers. We built a logistic regression analysis model to test the associations of frailty with the parameters that resulted in statistical differences in frail and non-frail patients.

Statistical significance was assessed for *p* values < 0.05.

Statistical analysis was conducted using IBM SPSS software (version 25).

## 3. Results

### 3.1. General Characteristics

The general characteristics of our population are shown in Table 1. Frail patients were older compared to non-frail ones (82 ± 6 vs. 76 ± 12, *p* = 0.009). There was a preponderance of men in the non-frail compared to the frail group (87% vs. 48%; *p* < 0.0001). There were no significant differences in eGFR, diabetes and BMI according to frailty status. Frail patients had higher MIS values (6 [4–11] vs. 4 [3–5], *p* < 0.0001). 

Similarly, frail patients also had lower pre-albumin and albumin levels compared to non-frail ones (pre-albumin: 27 ± 5 vs. 29 ± 5 mg/dL, *p* = 0.014 and albumin: 3.9 ± 0.3 vs. 4.1 ± 0.4 g/dL, *p* = 0.014). Serum concentrations of CRP and inflammatory cytokines did not differ between the two groups.

### 3.2. Correlations between Inflammatory Markers, Frailty and Malnutrition

Overall, the concentrations of inflammatory markers did not differ between frail and non-frail individuals (Table 1). Also, no difference was observed in terms of white blood cells in the blood between frail and non-frail patients (Table 1). However, some inflammatory markers were correlated with frailty domains (Table 2). In particular, weight loss was correlated with both CRP and MCP-1 (Rho 0.20, *p* = 0.041 and ρ 0.19, *p* = 0.041, respectively). Slowness was directly related to CRP (Rho 0.19, *p* = 0.043) and weakness showed a strong direct correlation with IL-6 (Rho 0.26, *p* = 0.001). No significant correlations were found between inflammatory markers, fatigue and low exercise capacity.

We also examined the correlations between soluble inflammatory markers and the MIS (Table 3). C-reactive protein was directly correlated with the MIS (CRP, Rho 0.198, *p* = 0.035). Furthermore, when comparing non-malnourished subjects (MIS < 3) with malnourished subjects (MIS > 8), we observed that MCP-1 was lower in patients with worse nutritional status (non-malnourished: 432.1 + 132.9 vs. malnourished: 407.8 + 165.2, *p* = 0.041).

After these initial observations, we also performed a multivariate linear regression analysis including TNFα, MCP-1, CRP and IL-6 to evaluate the possible independent association of systemic inflammatory markers with MIS. CRP remained independently and directly associated with MIS (B 0.266, *p* = 0.013).

**Figure 1:**
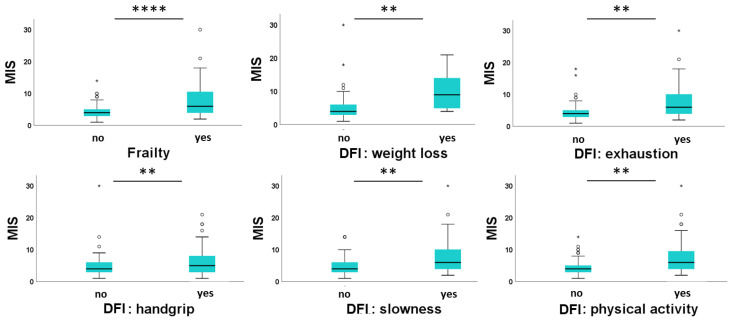
Box plot representative of MIS correlation with frailty and frailty criteria. Note: DFI, domain of frailty index; MIS, malnutrition–inflammation score. ° potential outliers, * extreme values ** *p* < 0.05, **** *p* < 0.001.

After evaluating the relationship between inflammatory markers, frailty and malnutrition, we directly examined the relationship between frailty and MIS (Figure 1). Overall, frail subjects had markedly higher MIS (*p* < 0.0001). In addition, MIS was higher in individuals with an impairment of any of the individual frailty domains (*p* < 0.001).

To determine, after various observations, which parameter was most strongly associated with an increased risk of frailty in our cohort, we performed a multivariable logistic regression analysis. We included MIS in addition to age, sex, Hb and pre-albumin. Higher MIS was the strongest independent factor associated with frailty (MIS OR 1.86, *p* = 0.002). Age and lower pre-albumin concentrations were also associated with increased odds of frailty, whereas male gender seemed to be protective in this context (Table 4). 

## 4. Discussion

The main finding of our study is that in older CKD patients, frailty is directly associated with malnutrition rather than with systemic inflammation. In particular, we found that MIS was independently associated with both overall frailty and individual frailty criteria, while none of the inflammatory cytokines were associated with overall frailty. 

Frailty was more prevalent in women rather than in men in our cohort, and no significant age difference was observed among genders in each subgroup. This is in line with previous reports [34,35].

We only observed some correlations between some inflammatory markers and specific domains of frailty: weight loss and slowness were associated with higher levels of MCP-1 and weakness was associated with higher concentrations of IL-6. Although our results do not support a direct association between systemic inflammation and frailty, we cannot exclude that inflammation may contribute to the onset and maintenance of malnutrition by modulating nutritional intake and favoring several specific catabolic pathways (especially in muscles) [36,37]. Overall, our results seem to indicate that in older CKD patients, systemic inflammation may contribute to the onset of frailty mainly by influencing nutritional status. In support of this hypothesis, it should be remembered that MIS is actually an integrated score of malnutrition and inflammation and proof of this is the fact that in our population, MIS is directly correlated with CRP values. In a previous paper we have demonstrated that, although older CKD patients had higher concentrations of inflammatory markers respect to healthy controls, sarcopenia was associated with malnutrition but not with systemic inflammation [22]. Therefore, malnourished individuals identified by the MIS represent the clinical phenotypes of individuals that are at high risk of becoming frail when they are not already.

Our current results are consistent with the literature; indeed, malnutrition is a well-known cause of frailty in CKD [18,19]. The etiology of malnutrition in CKD is composite: anorexia, uremic gastritis and polypharmacy may all contribute to reducing protein and energy intake [7,19,20,21,38]. A reduction in protein and energy intake causes muscle catabolism and reduced synthesis of muscle proteins, leading to sarcopenia and frailty [39,40]. Moreover, malnutrition is strongly associated with higher morbidity and mortality [39].

In CKD patients, systemic inflammation begins to rise progressively as kidney function declines. Uremic toxin accumulation, gut microbiota imbalance (translocation of endotoxins into the systemic circulation), and immune dysregulation (macrophages exposed to uremic toxins) contribute to the increased production of cytokines and acute phase proteins [41].

Our data are also consistent with what has been reported in the literature regarding the relationship between inflammation and malnutrition. The coexistence of malnutrition and inflammation in CKD patients is a well-recognized condition [20,42,43,44], inducing both protein degradation and the suppression of protein synthesis [45,46,47,48]. Furthermore, in a study performed in CKD patients, malnutrition was associated with dysfunctional neutrophil phagocytic activity which impairs immunity and favors the persistence of inflammation [49,50].

In both CKD and ESRD patients, both malnutrition and inflammation have been studied and evaluated separately regarding their association with frailty [51,52]. Instead, in our study, the associations between frailty, malnutrition and inflammation were assessed simultaneously in patients with CKD who were not yet on dialysis.In contrast to what has been described previously in the general population and in experimental models [53], we did not find any significant association between inflammatory markers and overall frailty. Only MCP-1, IL-6 and CRP were associated with some frailty domains. In patients on hemodialysis, inflammation is associated with frailty as well as with its worsening [54]. However, this was not observed in patients with better renal function. This may depend on the fact that either a lower persistence of inflammation or lower average concentrations of inflammatory markers may mitigate their influence on frailty. On the other hand, although we did not find any correlation with overall frailty, we found that specific inflammatory markers were associated with the impairment of some of its domains. These results may suggest that systemic inflammation may initially impair some of its defining domains like nutritional status (i.e., involuntary weight loss) and physical performance (walking speed) before inducing the clinical condition of full-blown frailty. In support of this hypothesis, we found that inflammatory markers are associated with malnutrition, a condition that is strongly associated with frailty in this population [39,55,56,57,58,59,60,61].

In our study, we did not focus on the various comorbidities and their potential correlation with renal and overall outcomes. This is because several studies have demonstrated that the various comorbidities included in scores like the Charlson Comorbidity Index are almost invariably present in the context of frailty [17,62]. Most importantly, it has been shown that frailty is a more powerful predictor of outcomes than individual comorbidities in several disease settings and in CKD patients, even when grouped in scoring systems like the Charlson Comorbidity Index [63,64,65].

Our study has several limitations. Firstly, the cross-sectional design does not allow us to attribute causality to the association between inflammatory marker levels, malnutrition and incident frailty in CKD patients. Moreover, we cannot demonstrate which determinant of frailty, between inflammation and malnutrition, developed first in singular individuals, and thus we cannot attribute a precise sequence of causality. Secondly, we did not collect any dietary or lifestyle information; therefore, we cannot attribute any specific role to these variables in determining malnutrition or frailty. Finally, our study is monocentric with a relatively small population. However, by using strict selection criteria, the monocentric nature of our study allowed us to reduce the potential sources of bias. In particular, we excluded patients who were inflamed or likely to be frail due to their clinical comorbidities and independently of CKD.

An important point of strength of our study is that we evaluated the relationship between frailty, malnutrition and inflammation in depth, looking not only at the overall frailty phenotype, but also at each individual domain of frailty. In addition, we looked at malnutrition not only with serum markers, but also with the MIS. The strict protocol for sampling and standardization also allowed us to obtain reliable and reproducible results.

To the best of our knowledge, this is the first time that a comprehensive assessment of the association between frailty, inflammation and malnutrition has been conducted in older CKD patients not in dialysis.

## 5. Conclusions

In conclusion, we found that in older CKD patients, frailty is associated with malnutrition–inflammation syndrome but not with systemic inflammatory cytokines. These findings underscore the importance of addressing malnutrition in order to prevent the onset of frailty in this population.

## Figures and Tables

**Table 1:** Comparison of general characteristics between frail and non-frail patients.

Variables	Non-Frail (*n* = 67)	Frail (*n* = 48)	*p*
General characteristics			
Age (years)	76 ± 12	82 ± 6	**0.009**
Males/Females, *n* (%)	58 (87)/9 (13)	23 (48)/25 (52)	**<0.0001**
Diabetes, *n* (%)	35 (52)	28 (58)	0.57
Hypertension, *n* (%)	60 (89)	44 (92)	0.76
Previous CV events, *n* (%)	33 (51)	29 (60)	0.20
BMI (kg/m^2^)	27.6 ± 4.1	28.6 ± 5.6	0.22
Metabolic characteristics			
eGFR (mL/min/1.73 m^2^)	25 ± 11	24 ± 10	0.63
Uric Acid (mg/dL)	6.1 ± 1.3	6.2 ± 1.7	0.87
HbA1c (mmol/mol)	44 ± 12	49 ± 13	0.089
Total Cholesterol (mg/dL)	165 ± 31	170 ± 44	0.47
HDL Cholesterol (mg/dL)	52 ± 15	55 ± 22	0.40
Triglycerides (mg/dL)	125 ± 55	132 ± 58	0.46
Iron (µg/dL)	76 ± 24	71 ± 24	0.33
Transferrin, (mg/dL)	229 ± 38	231 ± 42	0.80
Ferritin (mg/dL)	187 ± 206	174 ± 293	0.77
Hb (g/dL)	12.8 ± 1.4	12.0 ± 1.3	**0.002**
WBC (cells/uL)	6836 + 1309	6954 + 1754	0.774
Neutrophils (% of WBC)	61.7 + 7.2	61.3 + 7.6	0.841
Lymphocytes (% of WBC)	24.7 + 4.9	25.7 + 5.6	0.664
PTH (ng/L)	74 ± 44	81 ± 60	0.47
Vitamin D 25 OH (ng/mL)	30 ± 14	27.6 ± 19.3	0.39
Urinary protein excretion (g/24 h)	475 [199–1291]	450 [200–1144]	0.60
Nutritional parameters			
MIS	4 [3–5]	6 [4–11]	**<0.0001**
Albumin (g/dL)	4.1 ± 0.4	3.9 ± 0.3	**0.014**
Pre-albumin (mg/dL)	29 ± 5	27 ± 5	**0.014**
Inflammatory markers			
CRP, (mg/dL)	0.2 [0.09–0.43]	0.23 [0.12–0.42]	0.83
TNFα, (pg/mL)	13.6 [9.5–17.1]	14.1 [9.4–20.1]	0.47
IL-6, (pg/mL)	3.4 [1.6–5.6]	3.3 [1.6–5.2]	0.30
MCP-1, (pg/mL)	417 [332–500]	433 [308–528]	0.57

Note: BMI: body mass index; eGFR: estimated glomerular filtration rate; MIS: Malnutrition–Inflammation Score; HbA1c: glycated hemoglobin; HDL: high-density lipoprotein; Hb: hemoglobin; WBC: white blood cells; PTH: parathormone; CRP: C-reactive protein; TNFα: tumor necrosis factor alpha; MCP-1: monocyte chemoattractant protein-1; IL-6: interleukin 6; IL-10: interleukin 10. Data are expressed as mean + standard deviation or median [interquartile range] when appropriated. *p* values are intended for trends and values less than 0.05 are indicated in bold.

**Table 2:** Correlation analyses between inflammatory markers and frailty criteria (continuous variables).

Frailty Domain	Variables	Correlation Coefficient	*p*
	CRP, (mg/dL)	0.20 *	**0.041**
	TNFα, (pg/mL)	−0.14 ^+^	0.13
Weight Loss	MCP-1, (pg/mL)	0.19 ^+^	**0.041**
	Il-6, (pg/mL)	−0.01 *	0.91
	IL-10, (pg/mL)	−0.07 *	0.47
	CRP, (mg/dL)	0.12 *	0.20
	TNFα, (pg/mL)	−0.107 ^+^	0.26
Weakness	MCP-1, (pg/mL)	−0.006 ^+^	0.95
	Il-6, (pg/mL)	0.26 *	**0.001**
	IL-10, (pg/mL)	0.005 *	0.96
	CRP, (mg/dL)	0.19 *	**0.043**
	TNFα, (pg/mL)	0.001 ^+^	0.99
Slowness	MCP-1, (pg/mL)	−0.15 ^+^	0.12
	Il-6, (pg/mL)	−0.014 *	0.89
	IL-10, (pg/mL)	0.11 *	0.27

Note: CRP: C-reactive protein; TNFα: tumor necrosis factor alpha; MCP-1: monocyte chemotactic protein1; IL-6: interleukin 6; IL-10: interleukin 10. Correlation analyses were performed using Spearman (Rho) or Pearson (p) models when appropriated. *p* values are intended for trends and values less than 0.05 are indicated in bold. * Sperman model. ^+^ Pearson model.

**Table 3:** Correlation analysis between inflammatory markers and MIS.

Variables	Correlation Coefficient (Rho)	*p*
CRP, (mg/dL)	0.198	**0.035**
TNFα, (pg/mL)	0.042	0.65
MCP-1, (pg/mL)	−0.06	0.53
Il-6, (pg/mL)	−0.05	0.64
IL-10, (pg/mL)	0.12	0.15

Note: CRP: C-reactive protein; TNFα: tumor necrosis factor alpha; MCP-1: monocyte chemotactic protein-1; IL-6: interleukin 6; IL-10: interleukin 10. Spearman’s Rho correlation analyses were performed; correlation coefficients are expressed as Rho while *p* values are intended for trends and values less than 0.05 are indicated in bold.

**Table 4:** Multivariable analysis of factors associated with established frailty.

	Variables	OR	*p*
Frailty	Age (years)	1.17 (1.06–1.29)	**0.002**
	Sex (Male)	0.08 (0.01–0.63)	**0.016**
	MIS	1.86 (1.25–2.77)	**0.002**
	Pre-Albumin (mg/dL)	0.85 (0.65–0.96)	**0.026**
	Hemoglobin (g/dL)	1.313 (0.82–2.09)	0.252

Note: MIS: malnutrition–inflammation score; Hb: hemoglobin; all variables were considered together in the same multivariable model; odds ratios (ORs) are expressed as OR (95% confidence interval). *p* values <0.05 are in bold.

## Data Availability

The datasets analyzed for this study can be found in the OSF repository https://osf.io/vy4mp (accessed on 10 September 2023).
